# Experimental design and quantitative analysis of microbial community multiomics

**DOI:** 10.1186/s13059-017-1359-z

**Published:** 2017-11-30

**Authors:** Himel Mallick, Siyuan Ma, Eric A. Franzosa, Tommi Vatanen, Xochitl C. Morgan, Curtis Huttenhower

**Affiliations:** 1 Department of Biostatistics, Harvard T.H. Chan School of Public Health, Boston, MA 02115 USA; 2 Broad Institute of MIT and Harvard, Cambridge, MA 02142 USA; 3 Department of Microbiology and Immunology, The University of Otago, Dunedin, New Zealand

## Abstract

Studies of the microbiome have become increasingly sophisticated, and multiple sequence-based, molecular methods as well as culture-based methods exist for population-scale microbiome profiles. To link the resulting host and microbial data types to human health, several experimental design considerations, data analysis challenges, and statistical epidemiological approaches must be addressed. Here, we survey current best practices for experimental design in microbiome molecular epidemiology, including technologies for generating, analyzing, and integrating microbiome multiomics data. We highlight studies that have identified molecular bioactives that influence human health, and we suggest steps for scaling translational microbiome research to high-throughput target discovery across large populations.

## Introduction

Population-scale studies of the human microbiome now have at their disposal a remarkable range of culture-independent and other molecular and cellular biology technologies, but the identification of elements of the microbiome that are functionally important for human health remains challenging. This is in part due to the variety of tools available and the diversity of processes that they measure: microbial community composition [1–3], species and strain diversity [4–7], genomic elements [8, 9], transcription, translation, and metabolism [10–12], along with the corresponding human molecular processes in multiple epithelial, immune, and other cell types [13–15]. Research challenges also arise, however, at the intersection of microbial ecology and molecular epidemiology, as population-scale microbiome study designs and methods that adequately account for human variability, environmental exposures, and technical reproducibility are also still in the early stages of development [14, 16–18].

Existing technologies for population-scale microbiome studies share many similarities with molecular epidemiology techniques for human gene expression and genome-wide association studies [19, 20]. Human-associated microbial communities are most often profiled in terms of their composition, for example by sequencing the 16S ribosomal RNA (rRNA) genes to yield phylogenetic or taxonomic profiles (abbreviated here as 16S amplicon profiling) [21]. 16S and other amplicon-based technologies [22] are limited in their phylogenetic ranges; for example, 16S rRNA gene studies primarily target bacteria, with some crossover, whereas 18S or internal transcribed spacer (ITS) studies typically target fungi. Although highly sensitive, these technologies also suffer from contamination, amplification, and extraction biases [23]. A subset of these issues are shared by whole-community shotgun metagenomic sequencing approaches, which can further describe the functional genetic potential of the entire community, but do not tell us what portion of this genetic potential is actively transcribed or translated in any particular environment [24, 25]. Community metatranscriptomics, metabolomics, and metaproteomics techniques are emerging to link nucleotide sequence-based profiles to their bioactive products [26, 27], as are complementary technologies such as immunoglobulin A gene sequencing (IgA-seq), immunoprofiling, and human cell screening techniques to jointly profile microbial and human host activities [13, 28, 29]. When combined with culture-based microbial characterization [30], recent advances in the resulting experimental toolkit have greatly improved our ability to identify relevant components of host–microbiome interactions.

Translational applications of the microbiome at the population scale, however, require careful experimental, computational, and statistical considerations, combining lessons learned from earlier molecular epidemiology with challenges unique to microbiome profiling. First, the identification of relevant human or microbial cellular and molecular mechanisms requires sufficiently precise technologies; if bioactivity is due to a particular microbial strain or transcript, for example, it is unlikely to be identified by amplicon sequencing. Next, the identification of signals that are sufficiently reproducible for clinical actionability requires well-powered experimental designs and, ideally, meta-analysis among studies—both challenging for current microbiome protocols. Many environmental exposures and covariates, such as diet or medications, must also be measured because the microbiome (unlike the human genome) can both modify and be modified by these factors. Finally, appropriate computational and statistical methods must be used during analysis, as many standard approaches can be prone to surprising false positive or negative rates. In this review, we thus detail the current best practices in this field with respect to these challenges, delineate methods and computational tools (or lack thereof) for addressing these challenges, and discuss potential future directions for conducting integrated multiomics studies in microbiome molecular epidemiology.

## Microbial strain as the fundamental epidemiological unit for microbiome taxonomic profiles

It has become increasingly apparent that many, although not all, analyses of translational activities in the human microbiome will require the identification and characterization of microbial taxa at the strain level. Many current culture-independent tools profile microbial community membership by delineating genera or species, but microbial epidemiologists have long recognized that not all strains within a species are equally functional, particularly with respect to pathogenicity. For example, *Escherichia coli* may be neutral to the host, enterohemorrhagic [9], or probiotic [31], and epidemiologists have long employed methods such as serotyping, phage typing, or pulse gel electrophoresis to reveal and track the relationships between microbial strains within single species (as opposed to communities) of interest. Indeed, there is enormous genomic variation within *E. coli* alone; studies suggest a pangenome of well over 16,000 genes, with ~ 3000 gene families present in most strains and fewer than 2000 universal genes [32, 33]. While more comprehensively characterized for *Escherichia* than for other genera, this variability is not atypical of many microbial species.

Critically, such inter-strain variation has phenotypic consequences for human health, even in such well-studied organisms as *E. coli*. For instance, the probiotic strain *E. coli* Nissle was isolated during World War I due to its ability to confer resistance to *Shigella* upon its host [31], despite the close relationship of this strain to the uropathogenic strain CFT073 [34]. *Escherichia* is not unique among human commensals in having a large pangenome with a relatively small core. The *Staphylococcus aureus* pangenome is also approximately five times larger than its core genome [35], and this variation likewise has important consequences in differentiating commensal staphylococci from methicillin-resistant *S. aureus* (MRSA) [36]. Even gut commensals that are not traditionally associated with pathogenicity, such as *Bacteroides vulgatus* [6, 37], may show large intra-species genomic variation. Like those of better-characterized pathogens, these genomic differences within commensal microbe species may have consequences for the host; for example, not only was *Prevotella copri* recently correlated with new-onset rheumatoid arthritis, but specific gene differences among *P. copri* strains were also correlated with this phenotype [38].

**Fig. 1:**
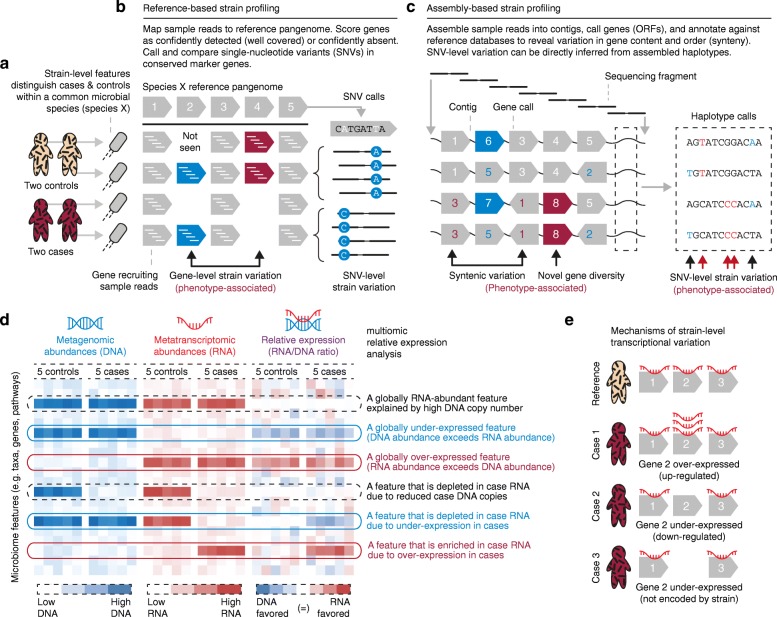
Strategies for detailed strain and molecular functional profiling of the microbiome in human population studies. **a** Culture-independent analysis methods can now identify members of the microbiome at the strain level using any of several related techniques. This is important in population studies as strains are often the functional units at which specific members of microbial communities can be causal in human health outcomes. **b** Among different approaches, reference-based methods can require less metagenomic sequence coverage (as little as ~ 1×), but are limited to identifying variation that is based on genes or single nucleotide variants (*SNVs*) related to available reference genomes. **c** Assembly-based methods can additionally resolve syntenic information across multiple markers at the cost of higher coverage (≥10×, Table 1). **d** ***,*** **e** Metatranscriptomic analysis, another emerging tool for characterizing microbiome function in human health, reveals over- or under-expression of microbial features with respect to their genomic content, both on **d** the population and **e** the individual level. *ORF* open reading frame

Although strain differences can have profound implications for human health, culture-independent tools have only recently begun to distinguish among strains during taxonomic profiling (Fig. 1a–c). For example, amplicon analyses are fundamentally limited in their ability to differentiate strains because critical functionality may arise from differences that occur outside of the otherwise-identical amplified gene regions (e.g., plasmids in *Escherichia* and *Shigella*). Both shotgun metagenomics and, when possible, 16S-based approaches can now be used to discriminate strains (Table 1), although both (especially the former) require care during such analyses. Most traditional operational taxonomic unit (OTU) clustering approaches for amplicon data, for example, differentiate only among taxa above some nucleotide identity threshold (e.g., 97% similarity). Likewise, metagenomic assembly protocols may intentionally avoid nucleotide-level variants. For 16S data, newer approaches [39–41] employ novel algorithms to distinguish between biological signal and sequencing error, and can discriminate small sequence differences corresponding to large phenotypic differences, such as sponge symbionts and their choice of host [39], or the specific ecological niches of human oral taxa [42]. Recent progress in developing bioinformatic tools further improves this resolution, revealing strain-level differentiation within the 16S region that can be as small as a single nucleotide [43–45].

**Table 1:** Tools for metagenomic strain analysis

Tool	Data type	Requires reference genome?	Method to resolve ambiguous reads	New strain detection	Recommended minimal coverage	Reference
Oligotyping	16S	–	–	–	–	[42]
Long-read 16S	16S	–	–	–	–	[119]
Minimum entropy decomposition	16S	–	–	–	–	[39]
OTU subpopulations	16S	–	–	–	–	[40]
LEA-seq	16S	–	–	–	–	[41]
DADA2	16S	No	Poisson modeling of sequence errors (the Divisive Amplicon Denoising Algorithm)	Yes	–	[43]
UNOISE2	16S	No	Abundance-based identification of sequencing errors	Yes	–	[44]
Deblur	16S	No	Abundance-based identification of sequencing errors	Yes	–	[45]
Megan	WGS	Yes	Lowest common ancestor algorithm	No	Not reported	[120]
GSMer	WGS	Yes	Unique strain-level markers (k-mers)	No	0.25×	[121]
WG-FAST	WGS	Yes	SNVs	No	3×	[122]
StrainPhlAn	WGS	Yes	SNVs within species-level marker genes	Yes	10×	[6]
PanPhlAn	WGS	Yes	Unique combinations of species-level marker genes	Yes	1×	[6]
MIDAS	WGS	Yes	Unique strain-level marker genes	Yes	Not reported	[37]
Sigma	WGS	Yes	Likelihood-based	No	0.027×	[123]
PathoScope	WGS	Yes	Likelihood-based	No	Less than 1×	[124]
ConStrains	WGS	Yes	Inferred haplotype-like SNP profiles	Yes	10×	[4]
LSA	WGS	De novo assembly	SVD-based K-mer clustering	Yes (not validated)	25 ~ 50×	[125]
CNV-based methods	WGS	Yes	Target gene or region copy number variation			[126]

*CNV* copy number variation, *LEA-Seq* low-error amplicon sequencing, *OTU* operational taxonomic unit, *SNP* single nucleotide polymorphism, *SNV* single-nucleotide variant, *SVD* singular-value decomposition, *WGS* whole-genome sequencing

Algorithms for strain identification from shotgun metagenomic sequences generally rely on one or both of two techniques: calling single nucleotide variants (SNVs, within a community or between community members and reference genomes) or identifying variable regions (such as gained or lost genomic elements; Table 1). Community SNV identification, like microbial isolate or human genetic profiling, requires sufficiently deep coverage (typically 10× or more) of every microbial strain to be differentiated [5], but can delineate closely related strains very precisely. SNVs can be assessed either extrinsically, with respect to one or more reference sequences (e.g., by mapping metagenomic sequences to that of reference and calling SNVs) [5], or intrinsically, by aligning sequences directly from one or more metagenomes and identifying SNVs among them [4]. Finally, as microbial strains often differ dramatically in their carriage of different core or pangenome elements or genomic islands (unlike most populations within eukaryote species [46]), strains can also be identified by the presence or absence of one or more genes or genomic regions [6]. This requires less sequencing depth (and is thus sensitive to less abundant members of a community), but can be more susceptible to noise and unable to delineate closely related strains.

Although strain identification, characterization, and phylogenetics are well-developed for microbial isolates [47], the use of culture-independent amplicon or metagenomic sequence data to perform such tasks is still in its infancy and can suffer from a variety of drawbacks. Amplicon methods in particular require variation to exist in the targeted region, and detecting the few variants that might exist in such short sequences requires extremely careful data generation and analysis protocols to distinguish biological from technical variation [39, 40]. Metagenomic strain identification is typically only accurate for the single most dominant strain of any one organism in complex communities, requiring extreme sequencing depths (e.g., tens to hundreds of gigabases) to differentiate secondary strains except when only one or a few organisms dominate [5]. Finally, as in other areas of microbial genomics, metagenomic strain identification is sensitive to the definition of a 'strain', which can vary from clonality at all genomic loci (possibly including plasmids), clonality at all sequenced locations (possibly only within an amplified region), or allowing some non-zero degree of nucleotide-level divergence [48].

## Metatranscriptomics enables characterization of context-specific, dynamic, biomolecular activity in microbial communities

Taxonomic profiling, at any level of resolution, is increasingly accompanied by functional profiling—pairing a community's organismal makeup with its gene and/or pathway catalog [9]. Metagenomic DNA sequencing, however, yields information only regarding the community's functional potential—which organisms, at what abundances, might be able to carry out which biological processes (and not necessarily which genes are being transcribed under current conditions). Metatranscriptomic RNA sequencing is arguably the first scalable, culture-independent technology to overcome this limitation, although its application to the human microbiome at an epidemiological scale still presents unique design and analysis challenges. Microbiome samples for metatranscriptomics must be collected in a manner that preserves RNA for sequencing, and they are (by definition) much more sensitive to the exact circumstances and timing of sample collection (Box 1) [17]. The associated protocols for nucleotide extraction are generally more challenging and sensitive to technical variability [49]. The resulting metatranscriptomes must generally be accompanied by paired metagenomes in order to allow interpretation of the data, otherwise changes in DNA copy number (i.e., microbial growth) cannot be differentiated from changes in transcriptional activity [24]. This is particularly true for amplicon-based rRNA metatranscriptomics, a proposed proxy for organismal growth or metabolic activity within a community [50]. In such settings, it is not yet clear how we could account for 16S rRNA gene copy number variation, differences in ribosomal transcription rates, or even the exact biological interpretation of 16S rRNA transcript abundances (as opposed to gene abundances as profiled by typical DNA amplicon sequencing).

By contrast, shotgun metatranscriptome studies provide biological information that complements metagenome studies, including detection of RNA viruses and quantification of rare but functional genes that might remain undetected in DNA-based metagenomic surveys [51] (Fig. 1d and e, and Table 2). Metatranscriptomic sequencing can also highlight the taxon- and strain-specific transcriptional activity of a community, providing a comprehensive overview of the functional ecology of the microbiome (Box 2). A typical metatranscriptomic study, such as a single-microbe RNA-seq study [52], consists of several steps, including: 1) transcript mapping and/or assembly; 2) annotation with functional and/or taxonomic information; 3) normalization; and 4) differential expression analysis. When processing reads, a metatranscriptomic analysis pipeline typically either maps reads to a reference genome or performs de novo assembly of the reads into transcript contigs. The first approach (mapping to a reference genome) is limited by the information in the reference database, whereas the second approach (de novo assembly) is limited by the difficulty of assembling long contigs of highly variable transcriptional coverage from complex metagenomes. Downstream bioinformatic analysis of metatranscriptomic expression profiles must further account for taxonomic composition variations and for technical biases associated with RNA-seq experiments. In particular, taxon-specific rescaling (RNA transcript abundance normalized to its DNA copy number) is a necessary step in order to ascertain whether apparent shifts in transcript levels are concordant with changes in taxon abundances. Finally, to conduct differential gene expression analysis post-normalization, off-the-shelf tools from single-organism RNA-seq can be used, some of which have already been adapted to microbial community settings [53].

**Table 2:** Tools for primary processing of metatranscriptomes

Tool	Assembly-based?	Requires reference genome?	Metatranscriptome-compatible?	Automatic statistics and/or figures?	Implementation	Comments/potential issues	Reference
Rockhopper	Yes	Yes	No	Yes	Java	Intended for isolates, not communities	[127]
HUMAnN	No	Yes	Yes	Yes	Python	–	[9]
Tuxedo	Yes	Yes	Yes	Yes	C++/R	–	[128]
IMP	Yes	No	Yes	Yes	Python/Docker	Relies on binning	[129]
SAMSA	Pairs only	No	Yes	Yes	MG-RAST	–	[130]
COMAN	No	No	Yes	Yes	Web-based/Python/R	Expression distribution of functional groups across phyla	[131]
IDBA-MT	Yes	No	No	No	C++	Only assembles	[132]
OASES	Yes	No	No	No	–	Only assembles	[133]
COGNIZER	No	No	No	No	C	Functional annotation framework for sequences	[134]
FMAP	No	No	Yes	Yes	Perl/R	–	[135]
MEGAN_CE	No	No	Yes	Yes	Java	All taxonomy or function assigned by BLASTing and binning reads	[136]
ShotMAP	No	No	Yes	Yes	Perl/R	Several tuning parameters	[137]

**Table 3:** Tools for feature-wise differential abundance analysis in microbial community taxonomic profiles

Tool	Counts or relative abundance	Normalization	Data transformation	Statistical model	Multivariable association	Random effects	*P* value calculation	Reference(s)
Metastats	Relative abundance	TSS	None	Nonparametric	No	No	Nonparametric *t*	[81]
LEfSe	Relative abundance	TSS	None	Nonparametric	No	No	Kruskal-Wallis	[80]
edgeR	Counts	TMM	None	Negative binomial	Yes	No	Fisher’s exact test	[76]
DESeq2	Counts	RLE	None	Negative binomial	Yes	No	Wald’s test	[77]
metagenomeSeq	Counts	CSS	Log	Zero-inflated Gaussian	Yes	No	Moderated *t* statistic	[74]
limma-voom	Counts	TMM	Voom	Gaussian	Yes	Yes	Moderated *t* statistic	[78]
MaAsLin	Relative abundance	TSS	Arcsine square root	Gaussian	Yes	Yes	Wald’s test	[75, 138]
ANCOM	Counts	Log ratio	None	Nonparametric	No	No	Mann-Whitney *U*	[82]
NBMM	Counts	None	None	Negative binomial	Yes	Yes	Wald’s test	[86]
ZINBMM	Counts	None	None	Zero-inflated negative binomial	Yes	Yes	Wald’s test	[103]
ZIBR	Relative abundance	TSS	No	Zero-inflated beta	Yes	Yes	Likelihood ratio	[85]

*CSS* cumulative sum scaling, *RLE* relative log expression, *TMM* trimmed mean by M-value, *TSS* total-sum scaling

## Box 1. Considerations for the collection of human microbiome specimens

The microbial ecology of body sites and niches across the human body is incredibly diverse, and studies of these different environments typically call for multiple different sample collection and storage methods. The initial restrictions placed on sample collection modalities are simply biophysical—a skin or oral sample may be swabbed, whereas saliva or oral rinse samples can be manipulated directly, and stool samples are often homogenized and/or aliquotted. Another main driver of sampling methodology is biomass, as quantities of bacteria vary tremendously in various parts of the human body, from 10^11^ bacteria on the skin to 10^14^ in the colon [54]. As a result, both total nucleic acid (DNA/RNA) yields and the proportion of extracted nucleic acid originating from the host are highly variable. The first experimental design considerations around sampling therefore include accessibility, degree of human (or other 'contaminant') nucleotides, and biomass.

At one extreme, stool is well-suited for metagenomics and metatranscriptomics because it is rarely subject to biomass limitations, and easily yields high quantities of microbial RNA and DNA with low host contamination (up to 75% of fecal mass is estimated to be bacterial [55]). By contrast, it is challenging to achieve DNA or RNA yields from skin swabs in the quantities required for typical shotgun sequencing library preparation. Finally, every human microbiome sample will contain some human DNA. In stool from healthy subjects, this comprises less than 1% of total DNA. The proportion of total DNA derived from the host is much higher in oral and skin (50–80%) samples [56]. For these reasons, 16S rRNA-based analysis rather than shotgun metaomic analysis may be beneficial for sample types such as skin or, particularly, tissue biopsies.

Once collected, human microbiome samples, especially those for population studies, must be stored and/or transported in a manner that is compatible with accurate profiling of the associated microbial communities. This typically entails snap freezing samples when possible (e.g., in a clinical setting), transporting them frozen (e.g., on ice), or employing a fixative that stops microbial growth and stabilizes nucleotides and/or other biomolecules. Multiple studies have assessed whether stabilization buffers can preserve microbial community DNA and RNA. One recent study examined the effects of temperature on oralpharangeal swabs and mock communities, and concluded that inadequate refrigeration caused community variation that was comparable to inter-individual variation [57].

Several studies have now examined the stability of stool under different fixative and storage regimes. Two recent studies both found that 95% ethanol and RNALater were comparable to immediate freezing at –80 °C for DNA preservation [24, 58]. Fewer than 5% of transcripts were affected by the choice of stabilization buffer [24]. Fecal microbiota transplantation (FMT) cards and DNA Genotek’s OmniGene commercial transport kit also induced less change in microbial communities than typical inter-individual variation. By contrast, preserving samples in 70% ethanol or storing at room temperature was associated with substantial changes in microbial community profiles, probably resulting from incomplete prevention of microbial growth [58].

For population studies, immediate freezing or shipping with ice packs may not be feasible. Microbiome samples of any type thus benefit from storage in a stabilization buffer, preferably with immediate homogenization. A variety of commercial collection kits are available to facilitate the collection of microbiome samples. DNA Genotek offers kits for a variety of body sites (oral, vaginal, sputum, and stool), some of which preserve RNA. Notably, in contrast to ethanol and RNALater, the preservative buffer does not need to be removed prior to kit-based sample extraction, although it may not be compatible with all molecular data types (e.g., metabolomics, proteomics). Other commercial entities have developed kits that can be used as part of an integrated microbiome profiling service. For example, uBiome offers a swab-based kit with a stabilization buffer that can be used for a variety of sample types (stool, genital, skin, and oral), which are typically employed during their own proprietary microbiome profiling. By contrast, the Biocollective offers a kit that allows the collection and cold shipping of an entire stool sample rather than a small aliquot. Given the range of options and constraints, a critical part of microbiome study design is to consider the cost of collection methods, the ability of these methods to provide sufficient biomass, their compatibility with a cohort’s postal or in-person logistics constraints, and the desired suite of downstream data generation modalities (possibly including microbial culture and/or gnotobiotics).

## Box 2. Ecological network inference

Individual species in microbial communities are not independent actors, and instead closely interact with one another to form a complex inter-dependent ecological network [59]. Microbial ecological networks provide insights into a wide range of interspecies and intercellular relationships including win–win (mutualism), lose–lose (competition), win–lose (parasitism, predation), win–zero (commensalism), and zero–lose (amensalism) [60]. Delineating these relationships is an important step toward understanding the overall function, structure, and dynamics of the microbial community.

Traditional approaches to defining these networks require the use of laboratory methods such as growth and co-culture assays and combinatorial labeling [61], which do not scale well to whole communities [62]. Computational approaches, conversely, are efficient but extremely prone to false positives because metaomic measurements are near-uniformly compositional [63] (in which case, for example, the expansion of a single microbe across samples induces spurious negative correlations with all other uniformly abundant microbes, because their relative abundances are simultaneously depressed). Recently, there has been considerable interest in the construction of compositionality-corrected microbial co-association networks [64–67]. These approaches vary in their ability to construct directed vs. undirected microbe–microbe interaction networks and range from simple correlation measures to more complex Gaussian graphical models, longitudinal dynamical systems models, and Bayesian networks (Table 4). Although a variety of network construction methods exist, methodologies for associating these microbial covariation and shift patterns with environmental parameters, clinical outcomes, and time gradients in human populations are currently lacking, making this a promising area for future research.

## Microbiome-associated metabolomics as an emerging opportunity to characterize bioactivity

Although several other culture-independent molecular methods are now joining metatranscriptomics for human microbiome profiling, non-targeted metabolomics may represent one of the most successful to date in explaining the mechanisms of bioactivity [26, 68]. This includes a range of nuclear magnetic resonance (NMR) and mass-spectrometry technologies for profiling small molecules from stool [26, 68], skin [69], circulating metabolites [70, 71], or coupled with other human-associated microbial communities. In many of these environments, it has been estimated that over 10% of small molecules may be of microbial origin or microbially modified [72], highlighting the need to associate specific microbial strains or genetic elements with the specific small molecules that, in turn, mediate human health phenotypes. The associated study designs have as yet seen limited application at the population scale, with some success stories highlighted below, and it remains to be seen which microbiome-associated metabolites are appropriate for predicting or modulating population health outcomes. The resulting data share similar strengths and weaknesses to metatranscriptomics; protocols are often still technically challenging, and while the resulting data may be more difficult to characterize at the molecular level, when possible they represent measurements that are often more directly causal (e.g., small molecules responsible for a specific bioactivity).

## Statistical questions, issues, and practice in modern epidemiological microbiome studies

**Fig. 2:**
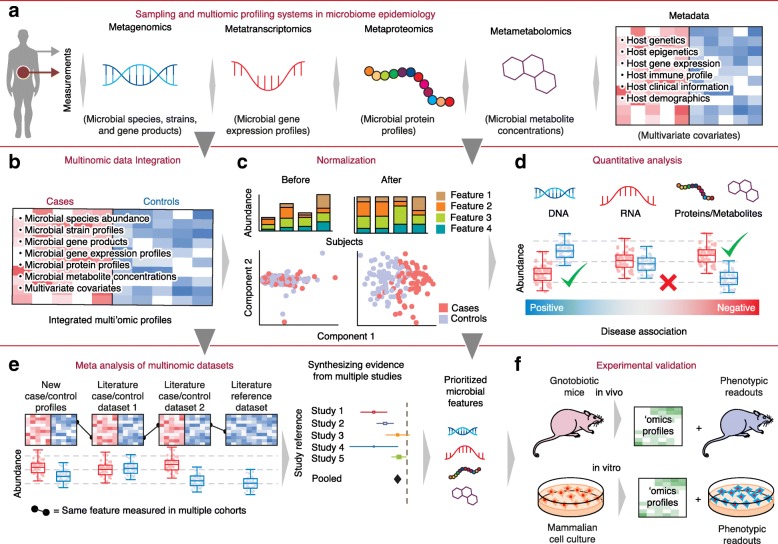
Microbiome molecular epidemiology. **a** Multiomic profiling of host and microbiota enables in-depth characterization of community properties from multiple culture-independent data types (including metagenomics, metatranscriptomics, metaproteomics, and metametabolomics) to address questions concerning the microbiome’s composition and function. **b** As in host-targeted molecular epidemiology, metagenomic and other metaomic data types can be integrated and associated with the available metadata to provide a comprehensive mechanistic understanding of the microbiome. **c** A wide range of early-stage data analysis choices can strongly affect microbial community data analysis, including the quality control of the raw data, the normalization of the raw data, choice of host and microbial features to extract, and algorithms to profile them. A hypothetical example of four taxonomic features is shown derived from four samples with differing metagenomic sequencing depths (*top*). Features with the same relative abundances may thus appear to be different on an absolute scale because larger sequencing depth can generate larger read counts (*top*). Normalization also corrects for potential batch effects and helps to preserve meaningful signal between cases and controls (*bottom*). Note that the precise methods used for global visualizations, such as the ordination method, can dramatically affect how the data are summarized, as can important parameters in the process, such as the (dis)similarity measures used to compare features or samples. **d** Within an individual study, the integration of multiple metaomic data types can provide stronger collective support for a hypothesis. Here, a hypothetical disease association is shown at the DNA, RNA, and protein or metabolite levels, providing a more complete picture of the disease pathogenesis. **e** When they differ between datasets, the strong technical effects that the choices mentioned above have on individual studies can impede multi-study meta-analyses, making this type of population-scale analysis difficult in the microbiome. When possible, the meta-analysis of host and microbial features with respect to shared phenotypes of interest can allow more confidence in prioritizing microbial taxa, gene products, or small molecules that have statistically significant roles in disease relative to covariates. **f** Finally, as with genome-wide association studies, it is critical to validate putative associations of top candidate microbial features with follow-up experimentation. In the microbiome, this can include studies involving animal models (such as gnotobiotic mice), mammalian cell systems, and/or microbial cultures

In all of these approaches—amplicon-based, shotgun sequencing, or other technologies—the persistent goal of microbiome epidemiology has been to determine whether and how microbial and molecular feature abundances are associated with the certain characteristics of the samples, such as donor health, disease status or outcome, donor dietary intake, donor medication, or environment (Fig. 2a–d). This translation of molecular epidemiology to the setting of the microbiome is challenging for several reasons. Among these is the technical nature of data associated with microbial communities, which typically consist of counts that have a compositional structure. That is, microbiome sample data (of most types) are frequently represented as vectors of fractional relative abundances (the total of all features in a sample sum to a value such as 1 or 100%). When typical statistical inference methods are used on compositional data, false positives result as a consequence of spurious correlation. This problem is exacerbated in population-scale microbiome studies by high data dimensionality (up to tens of thousands of samples containing potentially millions of microbial features), sparsity (made more challenging as the result of a mix of true zeros and undersampling events), and mean-variance dependency (variance of counts changes with the value of the mean) [63]. Failure to account for these specific characteristics of microbiome count data during statistical analysis can lead to strong biases in results; in particular, false positives outcomes are common, leading to irreproducible associations even (or especially) in large cohorts [73].

Several analysis methods have been developed to specifically address these problems in tests for differential feature abundance in the microbiome (Table 3 and Box 3). Virtually all of these methods rely on some form of normalization, and they differ primarily in the choice of the data transformation, statistical model, and null distribution (or equivalent) for *p* value calculation. For example, metagenomeSeq [74] takes raw read counts as input and accounts for possible biases using a zero-inflated Gaussian mixture model to integrate normalization and differential abundance analysis of log-counts. MaAsLin [75] uses a variance-stabilizing arcsine square root transformation to create continuous abundance profiles that can be analyzed by regular linear models. Apart from these community-specific tools, methods developed for differential expression analysis of similar RNA-seq data—such as edgeR [76], DESeq2 [77], and limma-voom [78]—have been adopted in microbiome research. These methods are typically based on a negative binomial statistical model of the normalized counts (with the exception of limma-voom, which applies an empirical Bayes linear model to the normalized counts) [53, 79]. Apart from these parametric approaches, several non-parametric alternatives have also been developed, such as LEfSe [80], Metastats [81], and ANCOM [82]. These methods make minimal assumptions about the data and estimate the null distribution for inference from ranks or from the observed data alone.

Normalization plays a crucial role in differential abundance analysis because variation in sequencing depth can make read counts incomparable across samples. Directly comparing read counts among samples with different sequencing depths may lead to the false conclusion that features are differentially abundant even when they have the same composition. In addition to simple total sum scaling (TSS) or rarefaction, this has led to the development of a variety of normalization approaches, such as trimmed mean of M-values (TMM) [83], relative log expression (RLE) [84], and cumulative sum scaling (CSS) [74], that aim to address the heteroscedasticity of the samples by variance stabilization and robustification or filtering [53]. Rarefaction is not ideal for many purposes because of its lack of statistical power and the existence of more appropriate methods [53], but it is fast and can be reasonably accurate in approximating a reliable normalization when necessary, especially given sufficient sequencing depth.

**Table 4:** Tools for compositionality-aware ecological network inference

Tool	Infers directed interaction?	Adjusts for covariates?	Reference(s)
CCREPE/ReBoot	No	No	[64]
SparCC	No	No	[65]
CCLasso	No	No	[66]
REBACCA	No	No	[139]
SPIEC-EASI	No	No	[67]
MENAP	No	No	[140]
MInt	No	Yes	[141]
MetaMIS	Yes	No	[142]
BioMiCo/BiomeNet	Yes	No	[143, 144]
MDSINE	Yes	No	[87]
DBN	Yes	No	[145]

Given the prominence of multivariate metadata in modern epidemiological cohorts, the availability of multivariable analysis tools is becoming increasingly important in the microbiome research community (Boxes 3 and 4). Some methods for differential abundance testing can only detect univariate associations, whereas other methods, such as edgeR, DESeq2, metagenomeSeq, limma-voom, and MaAsLin, can perform multivariable association. Future microbiome analytical tools must further leverage the hierarchical, spatial, and temporal nature of modern study designs, which typically result from repeated measurements across subjects, body sites, and time points. Several recent studies have taken initial steps to address one or both of these issues. One avenue of research aims to capture the correlation among repeated measurements by using random effects [75, 78, 85, 86]; other studies have relied on dynamical system or probabilistic spline modeling [87] of microbiome time-series data to study the temporal dynamics and stability of microbial ecosystems. Despite these innovations, the longitudinal modeling of microbiome data is still in its infancy, particularly in combination with multiple covariates in large human populations. There is a dearth of systematic studies aimed at the evaluation of multiple-covariate, repeated-measure methods for microbiome epidemiology, with no clear consensus to date. As microbiome data continue to accumulate, there is a pressing need for a rigorous comparison of these multivariable tools to help guide experimental designers and meta-analysts.

Many current microbiome epidemiology studies also use unsupervised models or visualizations to reveal structural patterns. Ordination is a particularly common visualization technique [21] that aims to plot samples in a low-dimensional space (usually no more than three axes) that also reflects their overall community similarities. This enables intuitive but rough inspection of strong signals in microbiome data (for example, an analyst might quickly identify samples with certain common characteristics that also have similar microbial compositions). Clustering analysis, also referred to as enterotyping or identifying community state types [88–90], is a related unsupervised technique for separating samples that have distinct profiles into different groups ('clusters'), and is appropriate only when distinct microbial sub-classes reliably exist in the data. Both methods have been heavily explored in high-dimensional biological datasets, such as gene expression and single-cell sequencing datasets, and while they can provide powerful tools for data overview and hypothesis generation, it is also important to recognize their limitations. First, both ordination and clustering analyses rely on a sample-against-sample dissimilarity (i.e., beta-diversity) matrix as input, and are thus sensitive to the choice of dissimilarity measure [73]. Second, as unsupervised approaches, both come with a wide variety of tunable parameters that are difficult to evaluate objectively. Third, for clustering analysis, distinguishing between discrete and continuous sample distribution patterns can be challenging when sample size is limited and/or signal is weak. Under such circumstances, quantitative examination of clustering strength is important to ensure that the identified clusters actually exist [89]. Finally, both methods are best suited to identifying the strongest patterns driven by population-level characteristics, both for microbiome data and in other ’omics settings [21]. To identify microbial associations with an outcome variable, supervised analysis [91] provides the resolution needed to identify patterns that might not be captured by the single strongest axis of variation, as well as rigorous, statistically justified quantification of such associations.

To this end, several families of omnibus test assess whether overall patterns of microbial variation in a community associate with covariates by some significance model (e.g., PERMANOVA [92], MiRKAT [93], ANOSIM [94]), typically with the ability to adjust for additional covariates. These tests are complementary to the supervised per-feature epidemiological association tests described above. They also take beta-diversity matrices as input, and they adopt statistically justified procedures to evaluate significance against the null hypothesis that covariates are not associated with overall microbiome composition. This is in contrast to the use of multiple individual tests for each microbial feature (species, clade, pathway, and so on) independently with respect to covariates, as described above. Similarly to ordination, the choice of dissimilarity measure can affect results, and some methods [93, 95] have correspondingly developed extensions to incorporate multiple metrics simultaneously in order to improve robustness. Another limitation of the omnibus testing methods is that, in some cases, only statistical significance (i.e., *p* values) are provided as output; newer methods aimed at assigning more interpretable effect sizes are under development [96]. Finally, omnibus testing procedures by definition do not identify what variation in a microbial community might be associated with an outcome of interest. Thus, although they may require smaller sample sizes than per-feature tests to be well-powered, they provide less actionable information as a result. Nevertheless, omnibus tests are an important accompaniment to unsupervised visualization in providing a quantitative model in support of qualitative data exploration by ordination.

## Box 3. Comparison of statistical methods for differential abundance analysis of microbiome data

Several studies have investigated the sensitivity and specificity of differential abundance tests (both omnibus and per-feature styles) for microbial communities using synthetic datasets [53, 73, 79, 97, 98]. No single best practice method that is appropriate for all circumstances has emerged, making the choice of an appropriate method for any given experimental setting a task for researchers with appropriate quantitative experience. In addition, it can be difficult for synthetic benchmark data to reflect accurately the statistical properties of microbiome data [67]. Hence, caution is needed when interpreting synthetic evaluations in the absence of an experimentally validated gold standard. With these caveats, some consistent findings have emerged from multiple comparison studies. First, special care should be taken when applying any methods to small sample sizes (e.g., < 50) [98]. Second, methods differ in their ability to handle count or count-like data versus relative abundances (Table 3). Finally, many of these tools have similar retrieval power for large datasets but can be too liberal in controlling the false discovery rate (FDR) [53, 73]. This probably reflects the fact that differential abundance detection largely depends on the accurate estimation of feature-specific variability, which remains difficult in sparse, compositional metagenomic datasets [73]. Besides statistical performance and computing efficiency, other issues to consider when choosing a tool include user-friendliness, ease of installation, and availability of high-quality documentation and tutorial data. As simulations typically rely on specific statistical distributions estimated primarily from technical replicates with minimal variation, comparisons using simulated datasets should be complemented with more practical comparisons in real datasets with true biological replicates.

## Box 4. Statistical terminologies—multivariate and multivariable associations

Microbiome data are inherently multivariate. This has led to the misleading conclusion that most published methods in microbiome literature are multivariate. Using terminology from classical statistics and regression analysis, most existing microbiome association methods can be categorized on the basis of how the outcome or target (also referred to as ‘dependent’ or ‘response’) variables of interest (left-hand side of a model equation) are modeled [99, 100].

‘Multivariate’ is the term used when two or more dependent variables are modeled simultaneously, an approach that is particularly suitable for relating the joint distribution of the responses to predictors. In statistics, ‘multivariable’ refers to approaches that include multiple explanatory variables or predictors (right hand side of the model equation) in a model (also known as ‘multiple regression’). ‘Univariate’ is a term used when one target variable is modeled at a time, completely ignoring interactions or correlations between dependent variables. Similarly, ‘univariable’ refers to models that include only one explanatory variable or predictor. Despite important differences between these paradigms, they are often used interchangeably in microbiome research. This imprecise reporting is also widespread in other disciplines such as public health, medicine, psychology, and political science [101, 102].

On the basis of the definitions provided above, most published analytical tools in microbiome epidemiology are essentially univariate (except PERMANOVA [92], which considers a distance matrix as (multivariate) dependent variable), and can be categorized as either simple (univariable) or multivariable (Table 3). Random effects models such as ZIBR [85], NBMM [86], ZINBMM [103], and MaAsLin [75] can be considered univariate multi-level or hierarchical models. These methods account for multiple responses per observation but consider each target variable (feature) separately. Other distance-based methods such as MiRKAT [93] are essentially multivariable methods as they usually consider the whole community profiles (or a mathematical function of the community distance matrix) as explanatory variables along with other covariates. Although interchangeable use of ‘multivariate’ and ‘multivariable’ seems to be only syntactic, we believe that achieving consensus on these terminologies will facilitate improved understanding and better communication among the next generation of microbiome researchers.

## Integration of studies needs to address confounding effects that are unique to microbiome data

Meta-analyses of microbiome features are becoming more desirable and common, particularly when scaled to large human populations in order to achieve reliability and power for translational findings (Fig. 2e and f). Meta-analysis [91] is, in general, the quantitative integration of findings from multiple studies, and it is crucial in any molecular 'omics field for verifying true, biological associations and improving power. Meta-analyses of most types of microbiome data face major challenges because of strong, batch- and study-specific biases that arise in most stages of data generation (sample collection, DNA extraction, PCR amplification, sequencing, and bioinformatics [17, 104]). Previous multi-cohort studies have confirmed the driving effect of study-specific protocols on the clustering of sample-specific microbial profiles (i.e., on population structure discovery). In the absence of active efforts to normalize protocols among meta-analyzed studies, the effects of these batch differences may be surpassed in strength only by a few extreme microbial phenotypes (such as body site of origin) and can easily mask even strong biological factors such as antibiotics usage and disease subtype [105].

Changes in protocol can thus heavily influence both overall community configuration and the abundances of individual features [23], making analyses such as meta-analytic differential abundance tests challenging. This does not, of course, prevent sufficiently strong effects from being observed across studies (for example, in inflammatory bowel disease patients). Although such issues are generally acknowledged in the microbiome research community, efforts to address them have been limited to date. From an experimental design point of view, sharing among studies one or more 'mock communities', comprised of reference material and/or pre-determined collections of microbial strains in known proportions, can provide a reference for identifying and estimating sources of bias [106]. Likewise, the publication of negative control sequencing results in a consistent manner would allow background subtraction and contaminant identification among studies. However, such controls need to be incorporated during the early stages of a study and cannot be added in retrospect. They have the potential to make meta-analysis much easier when included. Mock communities can also be technically challenging to generate and, of course, incur additional costs during data generation, but they are likely to be of high value if included systematically in multiple studies within and across projects.

To enable true meta-analysis of microbial community surveys, quantitative protocols to adjust for batch- and study-specific effects must be developed. For population structure identification and adjustment, additional steps are necessary to correct for and reduce such effects before comparing and aggregating samples from different studies. Existing popular methods in RNA-seq whole-transcriptome profiling—such as ComBat [107] and limma [108]—may be potential candidates, though they should be modified to account for the zero-inflated and compositional (or count) nature of microbial abundances. For single-feature differential abundance analysis, study-specific effects may alternatively be addressed by adopting a unified model with identically defined effect sizes, which can then be compared and combined across studies using existing proper statistical methods (for example, mixed-effects models [86, 109]). Another promising direction is high-dimensional predictive modeling techniques (that is, using subjects’ microbial profiles as predictors for outcomes of interests), such as random forests, neural networks, and support vector machines, which are often successful in reproducibly predicting phenotype across multiple cohorts [91, 110]. The results obtained to date suggest avenues by which discriminative machine-learning models can be applied in microbial community settings to robustly associate features across multiple studies with outcomes of interest.

## Conclusions

Like existing molecular epidemiology technologies, the translation of population studies of the human microbiome will require complex processes in order to achieve observational discovery, reproducibility across cohorts, and mechanistic validation (typically in models or in vitro). To date, a small number of studies have achieved this goal. For example, combining mouse models with a small cohort of 20 human subjects, Haiser and colleagues [111] built on decades of work linking *Eggerthella lenta* to inactivation of digoxin [112] to identify an operon that is expressed in a strain-specific manner in a subset of human microbiome carriers. As a further example, it has been shown that early-life exposure to distinct forms of taxon-specific lipopolysaccharide correlate with immune development and type 1 diabetes (T1D) risk, a result that was subsequently confirmed in mouse models (Box 5) [16]. Finally, in *Clostridium difficile* infection, models linking antibiotic exposure to bacterial species that are responsible for secondary bile acid synthesis in the gut have been successful in reducing recurrence [113]. In each of these cases, a combination of human population surveys with appropriate statistical modeling and mechanistic follow-up was able to identify specific bioactive microbes and, often, molecules. Further examples are emerging, particularly in the area of cancer immunotherapy, which can be dramatically modulated by the microbiome [114].

One of the outstanding gaps in translational population-scale microbiome studies is the lack of frameworks integrating host and microbiome functional properties at scale. For example, functional profiling of microbiome metagenomes and metatranscriptomes might be combined with cell-circuit reconstructions of immune cell subsets [115] and with electronic medical records for precision medicine. At the methodological level, few profiles of the microbiome have been carried out with scale and precision appropriate for advanced machine-learning tools such as causal inference and mediation analysis. Indeed, it is not yet clear which covariates should be collected to disambiguate cause from effect in the highly modifiable microbiome, particularly to facilitate risk-prediction models or clinical decision-making tools incorporating microbiome profiles. The microbiome has shown a remarkable combination of long-term persistence (e.g., strain retention for months or years [41, 116, 117]) with modifiability by a wide range of environmental factors (diet, pharmaceuticals, physical activity, age, and so on), making population structure and unobserved confounders a risk in large cohort studies.

Finally, human population studies provide a starting point for the follow-up characterization of microbial biochemical mechanisms, which can integrate characterization techniques such as culture-based physiology, microbial metabolism, co-culture, and interactions. Several of the most successful translational microbiome studies to date have—as in other areas of molecular epidemiology—begun with a population-level observation that was, eventually, traced back to one or more specific molecular mechanisms. In the case of the microbiome, this provides unique opportunities not only for prioritization of novel human drug targets, but also for the modulation of microbial activities by small molecules, diet or prebiotics, targeted probiotics, or engineered microbes or communities. To achieve these goals, studies of the microbiome must continue to refine the multiomic tools in the setting of population-scale epidemiology with rich study designs that can fully realize the therapeutic and diagnostic potential of the microbiome.

## Box 5. An integrative analysis of longitudinal microbiome multiomics: the DIABIMMUNE study

The DIABIMMUNE (Pathogenesis of Type 1 Diabetes—Testing the Hygiene Hypothesis) [118] study of the microbiome in the development of infant type 1 diabetes (T1D) is one example that incorporates many of the aspects of microbiome epidemiology discussed here. The DIABIMMUNE cohort includes newborn infants with genetic susceptibility to autoimmune disorders who were followed for 3 years with monthly stool sampling and collection of phenotype data through serum samples and questionnaires. This design was constructed to enable multiple types of microbiome analyses, such as tracking the longitudinal trajectories of the developing microbiomes, studying the implications of common early-life events (e.g., birth mode, weaning, introduction of solid foods, antibiotic courses) and case–control comparison between diseased and healthy children.

One of the study’s first analyses of the gut microbiome focused on early-life colonization and the development of islet autoimmunity and T1D [1]. The sub-cohort included four children with early onset T1D, seven children with T1D-associated autoantibodies, and 22 healthy controls. All subjects provided monthly stool samples, regardless of disease status, yielding a detailed view of microbiome structure and function during early development (including the transition to solid food). Strains in particular were subject-specific and retained for substantial periods of time, even during this active developmental window. In an early example of multiomic data integration, a subset of 214 serum and 104 stool samples were also profiled using untargeted mass spectrometry techniques, allowing covariation between metabolites and microbial taxa to be assessed statistically.

Another analysis within this study followed neonates from Finland, Estonia, and Russia, motivated by the disparate autoimmune prevalence between these three countries [16]. This began with 16S amplicon sequencing of > 1500 stool samples from 222 infants (74 per country), allowing the assessment of broad trends in microbiome development over time. These initial amplicon data were then used to select a representative set of 785 stool samples for metagenomic sequencing, which enabled deeper analyses including taxonomic and functional profiling, and strain tracking. All of these features were then amenable to linear mixed-effect modeling in order to identify aspects of the gut microbiome that covaried with phenotypes such as age, geography, early feeding, and mode of birth.

In this metagenomic sequencing study, a set of microbial products with geographically disparate abundances (and thus potentially associated with differential atopic and T1D outcomes) were identified computationally in tandem with potential source microbes. To verify their relevance in vitro, a subset (including lipopolysaccharide from several different microbial strains) was purified and screened against multiple different immune cell types. This allowed distinct structural and immunomodulatory properties to be identified, linking biochemical products to both source microbes and immune cellular phenotypes (e.g., cytokine production). Finally, a mouse model was used to show that these properties could, in turn, influence the outcome of interest, incidence of a model T1D phenotype.

## Abbreviations

rRNA: Ribosomal RNA
SNV: Single nucleotide variant
T1D: Type 1 diabetes

## References

[1] Kostic AD, Gevers D, Siljander H, Vatanen T, Hyotylainen T, Hamalainen AM (2015). The dynamics of the human infant gut microbiome in development and in progression toward type 1 diabetes. Cell Host Microbe.

[2] Yatsunenko T, Rey FE, Manary MJ, Trehan I, Dominguez-Bello MG, Contreras M (2012). Human gut microbiome viewed across age and geography. Nature.

[3] Costello EK, Lauber CL, Hamady M, Fierer N, Gordon JI, Knight R (2009). Bacterial community variation in human body habitats across space and time. Science.

[4] Luo C, Knight R, Siljander H, Knip M, Xavier RJ, Gevers D (2015). ConStrains identifies microbial strains in metagenomic datasets. Nat Biotechnol.

[5] Truong DT, Tett A, Pasolli E, Huttenhower C, Segata N (2017). Microbial strain-level population structure and genetic diversity from metagenomes. Genome Res.

[6] Scholz M, Ward DV, Pasolli E, Tolio T, Zolfo M, Asnicar F (2016). Strain-level microbial epidemiology and population genomics from shotgun metagenomics. Nat Methods.

[7] Human Microbiome Project Consortium (2012). Structure, function and diversity of the healthy human microbiome. Nature.

[8] Qin J, Li R, Raes J, Arumugam M, Burgdorf KS, Manichanh C (2010). A human gut microbial gene catalogue established by metagenomic sequencing. Nature.

[9] Abubucker S, Segata N, Goll J, Schubert AM, Izard J, Cantarel BL (2012). Metabolic reconstruction for metagenomic data and its application to the human microbiome. PLoS Comput Biol.

[10] Verberkmoes NC, Russell AL, Shah M, Godzik A, Rosenquist M, Halfvarson J (2009). Shotgun metaproteomics of the human distal gut microbiota. ISME J.

[11] Zeevi D, Korem T, Zmora N, Israeli D, Rothschild D, Weinberger A (2015). Personalized nutrition by prediction of glycemic responses. Cell.

[12] Suez J, Korem T, Zeevi D, Zilberman-Schapira G, Thaiss CA, Maza O (2014). Artificial sweeteners induce glucose intolerance by altering the gut microbiota. Nature.

[13] Schirmer M, Smeekens SP, Vlamakis H, Jaeger M, Oosting M, Franzosa EA (2016). Linking the human gut microbiome to inflammatory cytokine production capacity. Cell.

[14] Zhernakova A, Kurilshikov A, Bonder MJ, Tigchelaar EF, Schirmer M, Vatanen T (2016). Population-based metagenomics analysis reveals markers for gut microbiome composition and diversity. Science.

[15] Rooks MG, Garrett WS (2016). Gut microbiota, metabolites and host immunity. Nat Rev Immunol.

[16] Vatanen T, Kostic AD, d'Hennezel E, Siljander H, Franzosa EA, Yassour M (2016). Variation in microbiome LPS immunogenicity contributes to autoimmunity in humans. Cell.

[17] Sinha R, Abnet CC, White O, Knight R, Huttenhower C (2015). The microbiome quality control project: baseline study design and future directions. Genome Biol.

[18] Falony G, Joossens M, Vieira-Silva S, Wang J, Darzi Y, Faust K (2016). Population-level analysis of gut microbiome variation. Science.

[19] Boutros PC (2015). The path to routine use of genomic biomarkers in the cancer clinic. Genome Res.

[20] Ward LD, Kellis M (2012). Interpreting noncoding genetic variation in complex traits and human disease. Nat Biotechnol.

[21] Hamady M, Knight R (2009). Microbial community profiling for human microbiome projects: tools, techniques, and challenges. Genome Res.

[22] Underhill DM, Iliev ID (2014). The mycobiota: interactions between commensal fungi and the host immune system. Nat Rev Immunol.

[23] Brooks JP, Edwards DJ, Harwich MD, Rivera MC, Fettweis JM, Serrano MG (2015). The truth about metagenomics: quantifying and counteracting bias in 16S rRNA studies. BMC Microbiol.

[24] Franzosa EA, Morgan XC, Segata N, Waldron L, Reyes J, Earl AM (2014). Relating the metatranscriptome and metagenome of the human gut. Proc Natl Acad Sci U S A.

[25] Booijink CC, Boekhorst J, Zoetendal EG, Smidt H, Kleerebezem M, de Vos WM (2010). Metatranscriptome analysis of the human fecal microbiota reveals subject-specific expression profiles, with genes encoding proteins involved in carbohydrate metabolism being dominantly expressed. Appl Environ Microbiol.

[26] McHardy IH, Goudarzi M, Tong M, Ruegger PM, Schwager E, Weger JR (2013). Integrative analysis of the microbiome and metabolome of the human intestinal mucosal surface reveals exquisite inter-relationships. Microbiome.

[27] Grassl N, Kulak NA, Pichler G, Geyer PE, Jung J, Schubert S (2016). Ultra-deep and quantitative saliva proteome reveals dynamics of the oral microbiome. Genome Med.

[28] Palm NW, de Zoete MR, Cullen TW, Barry NA, Stefanowski J, Hao L (2014). Immunoglobulin A coating identifies colitogenic bacteria in inflammatory bowel disease. Cell.

[29] Geva-Zatorsky N, Sefik E, Kua L, Pasman L, Tan TG, Ortiz-Lopez A (2017). Mining the human gut microbiota for immunomodulatory organisms. Cell.

[30] Rajilic-Stojanovic M, de Vos WM (2014). The first 1000 cultured species of the human gastrointestinal microbiota. FEMS Microbiol Rev.

[31] Scaldaferri F, Gerardi V, Mangiola F, Lopetuso LR, Pizzoferrato M, Petito V (2016). Role and mechanisms of action of *Escherichia coli* Nissle 1917 in the maintenance of remission in ulcerative colitis patients: an update. World J Gastroenterol.

[32] Kaas RS, Friis C, Ussery DW, Aarestrup FM (2012). Estimating variation within the genes and inferring the phylogeny of 186 sequenced diverse *Escherichia coli* genomes. BMC Genomics.

[33] Salipante SJ, Roach DJ, Kitzman JO, Snyder MW, Stackhouse B, Butler-Wu SM (2015). Large-scale genomic sequencing of extraintestinal pathogenic *Escherichia coli* strains. Genome Res.

[34] Dobrindt U, Chowdary MG, Krumbholz G, Hacker J (2010). Genome dynamics and its impact on evolution of *Escherichia coli*. Med Microbiol Immunol.

[35] Bosi E, Monk JM, Aziz RK, Fondi M, Nizet V, Palsson BO (2016). Comparative genome-scale modelling of *Staphylococcus aureus* strains identifies strain-specific metabolic capabilities linked to pathogenicity. Proc Natl Acad Sci U S A.

[36] Diep BA, Gill SR, Chang RF, Phan TH, Chen JH, Davidson MG (2006). Complete genome sequence of USA300, an epidemic clone of community-acquired methicillin-resistant *Staphylococcus aureus*. Lancet.

[37] Nayfach S, Rodriguez-Mueller B, Garud N, Pollard KS (2016). An integrated metagenomics pipeline for strain profiling reveals novel patterns of bacterial transmission and biogeography. Genome Res.

[38] Scher JU, Sczesnak A, Longman RS, Segata N, Ubeda C, Bielski C (2013). Expansion of intestinal *Prevotella copri* correlates with enhanced susceptibility to arthritis. Elife.

[39] Eren AM, Morrison HG, Lescault PJ, Reveillaud J, Vineis JH, Sogin ML (2015). Minimum entropy decomposition: unsupervised oligotyping for sensitive partitioning of high-throughput marker gene sequences. ISME J.

[40] Tikhonov M, Leach RW, Wingreen NS (2015). Interpreting 16S metagenomic data without clustering to achieve sub-OTU resolution. ISME J.

[41] Faith JJ, Guruge JL, Charbonneau M, Subramanian S, Seedorf H, Goodman AL (2013). The long-term stability of the human gut microbiota. Science.

[42] Eren AM, Borisy GG, Huse SM, Mark Welch JL (2014). Oligotyping analysis of the human oral microbiome. Proc Natl Acad Sci U S A.

[43] Callahan BJ, McMurdie PJ, Rosen MJ, Han AW, Johnson AJ, Holmes SP (2016). DADA2: high-resolution sample inference from Illumina amplicon data. Nat Methods.

[44] Edgar RC. UNOISE2: improved error-correction for Illumina 16S and ITS amplicon sequencing. bioRxiv. 2016; doi: https://doi.org/10.1101/081257.

[45] Amir A, McDonald D, Navas-Molina JA, Kopylova E, Morton JT, Zech Xu Z, et al. Deblur rapidly resolves single-nucleotide community sequence patterns. mSystems. 2017;2. 10.1128/mSystems.00191-16 PMC5340863 28289731

[46] McInerney JO, McNally A, O'Connell MJ (2017). Why prokaryotes have pangenomes. Nat Microbiol.

[47] Loman NJ, Pallen MJ (2015). Twenty years of bacterial genome sequencing. Nat Rev Microbiol.

[48] Konstantinidis KT, Ramette A, Tiedje JM (2006). The bacterial species definition in the genomic era. Philos Trans R Soc Lond B Biol Sci.

[49] Giannoukos G, Ciulla DM, Huang K, Haas BJ, Izard J, Levin JZ (2012). Efficient and robust RNA-seq process for cultured bacteria and complex community transcriptomes. Genome Biol.

[50] Blazewicz SJ, Barnard RL, Daly RA, Firestone MK (2013). Evaluating rRNA as an indicator of microbial activity in environmental communities: limitations and uses. ISME J.

[51] Franzosa EA, Hsu T, Sirota-Madi A, Shafquat A, Abu-Ali G, Morgan XC, Huttenhower C (2015). Sequencing and beyond: integrating molecular 'omics' for microbial community profiling. Nat Rev Microbiol.

[52] Conesa A, Madrigal P, Tarazona S, Gomez-Cabrero D, Cervera A, McPherson A (2016). A survey of best practices for RNA-seq data analysis. Genome Biol.

[53] McMurdie PJ, Holmes S (2014). Waste not, want not: why rarefying microbiome data is inadmissible. PLoS Comput Biol.

[54] Sender R, Fuchs S, Milo R (2016). Revised estimates for the number of human and bacteria cells in the body. PLoS Biol.

[55] Stephen AM, Cummings JH (1980). The microbial contribution to human faecal mass. J Med Microbiol.

[56] Human Microbiome Project Consortium (2012). A framework for human microbiome research. Nature.

[57] Hang J, Desai V, Zavaljevski N, Yang Y, Lin X, Satya RV (2014). 16S rRNA gene pyrosequencing of reference and clinical samples and investigation of the temperature stability of microbiome profiles. Microbiome.

[58] Song SJ, Amir A, Metcalf JL, Amato KR, Xu ZZ, Humphrey G, Knight R. Preservation methods differ in fecal microbiome stability, affecting suitability for field studies. mSystems 2016;1. 10.1128/mSystems.00021-16 PMC5069758 27822526

[59] Faust K, Raes J (2012). Microbial interactions: from networks to models. Nat Rev Microbiol.

[60] Mitri S, Foster KR (2013). The genotypic view of social interactions in microbial communities. Annu Rev Genet.

[61] Tan J, Zuniga C, Zengler K (2015). Unraveling interactions in microbial communities—from co-cultures to microbiomes. J Microbiol.

[62] Yu Z, Krause SM, Beck DA, Chistoserdova L (2016). A synthetic ecology perspective: how well does behavior of model organisms in the laboratory predict microbial activities in natural habitats?. Front Microbiol.

[63] Tsilimigras MC, Fodor AA (2016). Compositional data analysis of the microbiome: fundamentals, tools, and challenges. Ann Epidemiol.

[64] Faust K, Sathirapongsasuti JF, Izard J, Segata N, Gevers D, Raes J, Huttenhower C (2012). Microbial co-occurrence relationships in the human microbiome. PLoS Comput Biol.

[65] Friedman J, Alm EJ (2012). Inferring correlation networks from genomic survey data. PLoS Comput Biol.

[66] Fang H, Huang C, Zhao H, Deng M (2015). CCLasso: correlation inference for compositional data through Lasso. Bioinformatics.

[67] Kurtz ZD, Muller CL, Miraldi ER, Littman DR, Blaser MJ, Bonneau RA (2015). Sparse and compositionally robust inference of microbial ecological networks. PLoS Comput Biol.

[68] Wu GD, Compher C, Chen EZ, Smith SA, Shah RD, Bittinger K (2016). Comparative metabolomics in vegans and omnivores reveal constraints on diet-dependent gut microbiota metabolite production. Gut.

[69] Bouslimani A, Porto C, Rath CM, Wang M, Guo Y, Gonzalez A (2015). Molecular cartography of the human skin surface in 3D. Proc Natl Acad Sci U S A.

[70] Org E, Blum Y, Kasela S, Mehrabian M, Kuusisto J, Kangas AJ (2017). Relationships between gut microbiota, plasma metabolites, and metabolic syndrome traits in the METSIM cohort. Genome Biol.

[71] Ashrafian H, Li JV, Spagou K, Harling L, Masson P, Darzi A (2014). Bariatric surgery modulates circulating and cardiac metabolites. J Proteome Res.

[72] Wikoff WR, Anfora AT, Liu J, Schultz PG, Lesley SA, Peters EC, Siuzdak G (2009). Metabolomics analysis reveals large effects of gut microflora on mammalian blood metabolites. Proc Natl Acad Sci U S A.

[73] Thorsen J, Brejnrod A, Mortensen M, Rasmussen MA, Stokholm J, Al-Soud WA (2016). Large-scale benchmarking reveals false discoveries and count transformation sensitivity in 16S rRNA gene amplicon data analysis methods used in microbiome studies. Microbiome.

[74] Paulson JN, Stine OC, Bravo HC, Pop M (2013). Differential abundance analysis for microbial marker-gene surveys. Nat Methods.

[75] Morgan XC, Tickle TL, Sokol H, Gevers D, Devaney KL, Ward DV (2012). Dysfunction of the intestinal microbiome in inflammatory bowel disease and treatment. Genome Biol.

[76] Robinson MD, McCarthy DJ, Smyth GK (2010). edgeR: a Bioconductor package for differential expression analysis of digital gene expression data. Bioinformatics.

[77] Love MI, Huber W, Anders S (2014). Moderated estimation of fold change and dispersion for RNA-seq data with DESeq2. Genome Biol.

[78] Law CW, Chen Y, Shi W, Smyth GK (2014). voom: precision weights unlock linear model analysis tools for RNA-seq read counts. Genome Biol.

[79] Jonsson V, Osterlund T, Nerman O, Kristiansson E (2016). Statistical evaluation of methods for identification of differentially abundant genes in comparative metagenomics. BMC Genomics.

[80] Segata N, Izard J, Waldron L, Gevers D, Miropolsky L, Garrett WS, Huttenhower C (2011). Metagenomic biomarker discovery and explanation. Genome Biol.

[81] White JR, Nagarajan N, Pop M (2009). Statistical methods for detecting differentially abundant features in clinical metagenomic samples. PLoS Comput Biol.

[82] Mandal S, Van Treuren W, White RA, Eggesbo M, Knight R, Peddada SD (2015). Analysis of composition of microbiomes: a novel method for studying microbial composition. Microb Ecol Health Dis.

[83] Robinson MD, Oshlack A (2010). A scaling normalization method for differential expression analysis of RNA-seq data. Genome Biol.

[84] Anders S, Huber W (2010). Differential expression analysis for sequence count data. Genome Biol.

[85] Chen EZ, Li H (2016). A two-part mixed-effects model for analyzing longitudinal microbiome compositional data. Bioinformatics.

[86] Zhang X, Mallick H, Tang Z, Zhang L, Cui X, Benson AK, Yi N (2017). Negative binomial mixed models for analyzing microbiome count data. BMC Bioinformatics.

[87] Bucci V, Tzen B, Li N, Simmons M, Tanoue T, Bogart E (2016). MDSINE: Microbial Dynamical Systems INference Engine for microbiome time-series analyses. Genome Biol.

[88] Arumugam M, Raes J, Pelletier E, Le Paslier D, Yamada T, Mende DR (2011). Enterotypes of the human gut microbiome. Nature.

[89] Koren O, Knights D, Gonzalez A, Waldron L, Segata N, Knight R (2013). A guide to enterotypes across the human body: meta-analysis of microbial community structures in human microbiome datasets. PLoS Comput Biol.

[90] Gajer P, Brotman RM, Bai G, Sakamoto J, Schutte UM, Zhong X (2012). Temporal dynamics of the human vaginal microbiota. Sci Transl Med.

[91] Pasolli E, Truong DT, Malik F, Waldron L, Segata N (2016). Machine learning meta-analysis of large metagenomic datasets: tools and biological insights. PLoS Comput Biol.

[92] Anderson MJ (2001). A new method for non‐parametric multivariate analysis of variance. Austral Ecol.

[93] Zhao N, Chen J, Carroll IM, Ringel-Kulka T, Epstein MP, Zhou H (2015). Testing in microbiome-profiling studies with MiRKAT, the Microbiome Regression-Based Kernel Association Test. Am J Hum Genet.

[94] Clarke KR (1993). Non‐parametric multivariate analyses of changes in community structure. Aust J Ecol.

[95] Tang ZZ, Chen G, Alekseyenko AV (2016). PERMANOVA-S: association test for microbial community composition that accommodates confounders and multiple distances. Bioinformatics.

[96] Randolph TW, Zhao S, Copeland W, Hullar M, Shojaie A. Kernel-Penalized regression for analysis of microbiome data. arXiv 2015;arXiv:151100297. 10.1214/17-AOAS1102 PMC6138053 30224943

[97] Jonsson V, Osterlund T, Nerman O, Kristiansson E (2017). Variability in metagenomic count data and its influence on the identification of differentially abundant genes. J Comput Biol.

[98] Weiss S, Xu ZZ, Peddada S, Amir A, Bittinger K, Gonzalez A (2017). Normalization and microbial differential abundance strategies depend upon data characteristics. Microbiome.

[99] Neter J, Kutner MH, Nachtsheim CJ, Wasserman W (1996). Applied linear statistical models.

[100] Olive DJ, Olive DJ (2017). Multivariate linear regression. Linear regression.

[101] Hidalgo B, Goodman M (2013). Multivariate or multivariable regression?. Am J Public Health.

[102] Tsai AC (2013). Achieving consensus on terminology describing multivariable analyses. Am J Public Health.

[103] Fang R, Wagner BD, Harris JK, Fillon SA (2016). Zero-inflated negative binomial mixed model: an application to two microbial organisms important in oesophagitis. Epidemiol Infect.

[104] Brooks JP (2016). Challenges for case-control studies with microbiome data. Ann Epidemiol.

[105] Lozupone CA, Stombaugh J, Gonzalez A, Ackermann G, Wendel D, Vazquez-Baeza Y (2013). Meta-analyses of studies of the human microbiota. Genome Res.

[106] Bokulich NA, Rideout JR, Mercurio WG, Shiffer A, Wolfe B, Maurice CF, et al. mockrobiota: a public resource for microbiome bioinformatics benchmarking. mSystems 2016;1. 10.1128/mSystems.00062-16 PMC5080401 27822553

[107] Johnson WE, Li C, Rabinovic A (2007). Adjusting batch effects in microarray expression data using empirical Bayes methods. Biostatistics.

[108] Ritchie ME, Phipson B, Wu D, Hu Y, Law CW, Shi W, Smyth GK (2015). limma powers differential expression analyses for RNA-sequencing and microarray studies. Nucleic Acids Res.

[109] Romero R, Hassan SS, Gajer P, Tarca AL, Fadrosh DW, Nikita L (2014). The composition and stability of the vaginal microbiota of normal pregnant women is different from that of non-pregnant women. Microbiome.

[110] Ananthakrishnan AN, Luo C, Yajnik V, Khalili H, Garber JJ, Stevens BW (2017). Gut microbiome function predicts response to anti-integrin biologic therapy in inflammatory bowel diseases. Cell Host Microbe.

[111] Haiser HJ, Gootenberg DB, Chatman K, Sirasani G, Balskus EP, Turnbaugh PJ (2013). Predicting and manipulating cardiac drug inactivation by the human gut bacterium *Eggerthella lenta*. Science.

[112] Saha JR, Butler VP, Neu HC, Lindenbaum J (1983). Digoxin-inactivating bacteria: identification in human gut flora. Science.

[113] Buffie CG, Bucci V, Stein RR, McKenney PT, Ling L, Gobourne A (2015). Precision microbiome reconstitution restores bile acid mediated resistance to *Clostridium difficile*. Nature.

[114] Vetizou M, Pitt JM, Daillere R, Lepage P, Waldschmitt N, Flament C (2015). Anticancer immunotherapy by CTLA-4 blockade relies on the gut microbiota. Science.

[115] Yosef N, Regev A (2016). Writ large: genomic dissection of the effect of cellular environment on immune response. Science.

[116] Schloissnig S, Arumugam M, Sunagawa S, Mitreva M, Tap J, Zhu A (2013). Genomic variation landscape of the human gut microbiome. Nature.

[117] Franzosa EA, Huang K, Meadow JF, Gevers D, Lemon KP, Bohannan BJ, Huttenhower C (2015). Identifying personal microbiomes using metagenomic codes. Proc Natl Acad Sci U S A.

[118] Peet A, Kool P, Ilonen J, Knip M, Tillmann V, Group DS (2012). Birth weight in newborn infants with different diabetes-associated HLA genotypes in three neighbouring countries: Finland, Estonia and Russian Karelia. Diabetes Metab Res Rev.

[119] Franzen O, Hu J, Bao X, Itzkowitz SH, Peter I, Bashir A (2015). Improved OTU-picking using long-read 16S rRNA gene amplicon sequencing and generic hierarchical clustering. Microbiome.

[120] Huson DH, Auch AF, Qi J, Schuster SC (2007). MEGAN analysis of metagenomic data. Genome Res.

[121] Tu Q, He Z, Zhou J (2014). Strain/species identification in metagenomes using genome-specific markers. Nucleic Acids Res.

[122] Sahl JW, Schupp JM, Rasko DA, Colman RE, Foster JT, Keim P (2015). Phylogenetically typing bacterial strains from partial SNP genotypes observed from direct sequencing of clinical specimen metagenomic data. Genome Med.

[123] Ahn TH, Chai J, Pan C (2015). Sigma: strain-level inference of genomes from metagenomic analysis for biosurveillance. Bioinformatics.

[124] Francis OE, Bendall M, Manimaran S, Hong C, Clement NL, Castro-Nallar E (2013). Pathoscope: species identification and strain attribution with unassembled sequencing data. Genome Res.

[125] Cleary B, Brito IL, Huang K, Gevers D, Shea T, Young S, Alm EJ (2015). Detection of low-abundance bacterial strains in metagenomic datasets by eigengenome partitioning. Nat Biotechnol.

[126] Greenblum S, Carr R, Borenstein E (2015). Extensive strain-level copy-number variation across human gut microbiome species. Cell.

[127] McClure R, Balasubramanian D, Sun Y, Bobrovskyy M, Sumby P, Genco CA (2013). Computational analysis of bacterial RNA-Seq data. Nucleic Acids Res.

[128] Ghosh S, Chan CK (2016). Analysis of RNA-Seq data using TopHat and Cufflinks. Methods Mol Biol.

[129] Narayanasamy S, Jarosz Y, Muller EE, Heintz-Buschart A, Herold M, Kaysen A (2016). IMP: a pipeline for reproducible reference-independent integrated metagenomic and metatranscriptomic analyses. Genome Biol.

[130] Westreich ST, Korf I, Mills DA, Lemay DG (2016). SAMSA: a comprehensive metatranscriptome analysis pipeline. BMC Bioinformatics.

[131] Ni Y, Li J, Panagiotou G (2016). COMAN: a web server for comprehensive metatranscriptomics analysis. BMC Genomics.

[132] Leung HC, Yiu SM, Parkinson J, Chin FY (2013). IDBA-MT: de novo assembler for metatranscriptomic data generated from next-generation sequencing technology. J Comput Biol.

[133] Schulz MH, Zerbino DR, Vingron M, Birney E (2012). Oases: robust de novo RNA-seq assembly across the dynamic range of expression levels. Bioinformatics.

[134] Bose T, Haque MM, Reddy C, Mande SS (2015). COGNIZER: a framework for functional annotation of metagenomic datasets. PLoS One.

[135] Kim J, Kim MS, Koh AY, Xie Y, Zhan X (2016). FMAP: Functional Mapping and Analysis Pipeline for metagenomics and metatranscriptomics studies. BMC Bioinformatics.

[136] Huson DH, Beier S, Flade I, Gorska A, El-Hadidi M, Mitra S (2016). MEGAN community edition—interactive exploration and analysis of large-scale microbiome sequencing data. PLoS Comput Biol.

[137] Nayfach S, Bradley PH, Wyman SK, Laurent TJ, Williams A, Eisen JA (2015). Automated and accurate estimation of gene family abundance from shotgun metagenomes. PLoS Comput Biol.

[138] Morgan XC, Kabakchiev B, Waldron L, Tyler AD, Tickle TL, Milgrom R (2015). Associations between host gene expression, the mucosal microbiome, and clinical outcome in the pelvic pouch of patients with inflammatory bowel disease. Genome Biol.

[139] Ban Y, An L, Jiang H (2015). Investigating microbial co-occurrence patterns based on metagenomic compositional data. Bioinformatics.

[140] Deng Y, Jiang YH, Yang Y, He Z, Luo F, Zhou J (2012). Molecular ecological network analyses. BMC Bioinformatics.

[141] Biswas S, McDonald M, Lundberg DS, Dangl JL, Jojic V (2016). Learning microbial interaction networks from metagenomic count data. J Comput Biol.

[142] Shaw GT, Pao YY, Wang D (2016). MetaMIS: a metagenomic microbial interaction simulator based on microbial community profiles. BMC Bioinformatics.

[143] Shafiei M, Dunn KA, Boon E, MacDonald SM, Walsh DA, Gu H, Bielawski JP (2015). BioMiCo: a supervised Bayesian model for inference of microbial community structure. Microbiome.

[144] Shafiei M, Dunn KA, Chipman H, Gu H, Bielawski JP (2014). BiomeNet: a Bayesian model for inference of metabolic divergence among microbial communities. PLoS Comput Biol.

[145] McGeachie MJ, Sordillo JE, Gibson T, Weinstock GM, Liu YY, Gold DR (2016). Longitudinal prediction of the infant gut microbiome with dynamic Bayesian networks. Sci Rep.

